# CaSR regulates SLC26A6 expression via the PKA-FOXO4 signaling axis to promote experimental calcium oxalate kidney stone formation in rats

**DOI:** 10.3389/fcell.2026.1746423

**Published:** 2026-03-20

**Authors:** Lifeng Gan, Wei Li, Peiyue Luo, Fangtao Zhang, Haidong Zhong, Minggui Xie, Yiran Lu, Biao Qian, Liying Zheng

**Affiliations:** 1 The First Clinical College, Gannan Medical University, Ganzhou, Jiangxi, China; 2 Department of Urology, The First Affiliated Hospital of Gannan Medical University, Ganzhou, Jiangxi, China; 3 Key Laboratory of Urology and Andrology of Ganzhou, Ganzhou, Jiangxi, China; 4 The Chaozhou Central Hospital, Chaozhou, Guangdong, China; 5 Department of Graduate, The First Affiliated Hospital of Gannan Medical University, Ganzhou, Jiangxi, China

**Keywords:** CaSR, FOXO4, kidney stones, molecular mechanism, PKA, SLC26A6

## Abstract

**Background/Objective:**

Kidney stones are a globally prevalent urological disease, with calcium oxalate stones being the most common type. Their pathogenesis is complex, and postoperative recurrence rates are high. The calcium-sensing receptor (CaSR) and solute carrier family 26 member 6 (SLC26A6) play key roles in hypercalciuria and hyperoxaluria, respectively. However, the intrinsic relationship and regulatory mechanism between them in kidney stone formation remain unclear. This study aims to investigate whether CaSR regulates SLC26A6 expression through a specific signaling pathway, thereby playing a role in experimental calcium oxalate kidney stone formation in rats.

**Methods:**

*In vivo*, a calcium oxalate kidney stone model was established in Wistar rats by intragastric administration of 1% ethylene glycol (E.G.,) and 1% ammonium chloride. Rats were divided into six groups: blank control (NC), E.G., model (E.G.,), CaSR agonist (CaSR-a), CaSR inhibitor (CaSR-i), protein kinase A inhibitor (PKA-i), and Forkhead box protein O4 inhibitor (FOXO4-i) groups. Urinary calcium and oxalate levels were measured. Kidney crystal formation was observed via Hematoxylin and Eosin (HE) staining and Pizzolato’s staining. Protein and mRNA expression of CaSR, p-protein kinase A (PKA) substrate, p-FOXO4 (Thr451), and SLC26A6 in kidney tissues were detected by Western blotting, immunohistochemistry, and Real-Time quantitative PCR (RT-qPCR). *In vitro*, rat renal tubular epithelial cells (NRK-52E) were intervened with calcium oxalate monohydrate (COM) crystals and treated with agonists or inhibitors of CaSR, PKA, and Forkhead box protein O4 (FOXO4). Pathway-related protein expression was detected by Western blotting. A dual-luciferase reporter gene assay was used to validate the transcriptional regulation of the SLC26A6 promoter by FOXO4.

**Results:**

Compared to the NC group, the, E.G., group showed significantly increased urinary calcium and oxalate concentrations, increased renal crystal deposition, and upregulated expression of CaSR, p-PKA substrate, p-FOXO4, and SLC26A6. Activating CaSR (CaSR-a group) further exacerbated these phenomena, whereas inhibiting CaSR (CaSR-i group), PKA (PKA-i group), or FOXO4 (FOXO4-i group) significantly alleviated crystal formation and reduced SLC26A6 expression. Cell experiments confirmed that activating CaSR enhanced PKA and FOXO4 phosphorylation and SLC26A6 expression; activating PKA enhanced FOXO4 phosphorylation and SLC26A6 expression; activating FOXO4 upregulated SLC26A6 expression. The dual-luciferase reporter gene assay showed that FOXO4 functionally regulates SLC26A6 promoter and regulates its transcriptional activity.

**Conclusion:**

During the formation of experimental calcium oxalate kidney stones in rats, CaSR activation promotes PKA-mediated FOXO4 phosphorylation, leading to upregulation of SLC26A6 expression through transcriptional mechanisms. This signaling axis promotes urinary oxalate excretion and contributes to kidney stone formation. This study reveals the potential role of the novel CaSR-PKA-FOXO4-SLC26A6 signaling pathway in the pathogenesis of kidney stones.

## Introduction

Kidney stones are a urological disease with high global prevalence and prominent recurrence rates, imposing a heavy health and economic burden on patients and society (Hill et al., 2022; Scales et al., 2012; Pearle et al., 2005). Among them, calcium oxalate stones account for over 80% of all kidney stone types. The core of their formation lies in the supersaturation of calcium oxalate in urine, with hypercalciuria and hyperoxaluria being the two most critical risk factors leading to this supersaturated state (Coe et al., 2005; Sancak et al., 2015). Studies have shown that the main mechanisms by which hyperuricemia promotes kidney stone formation include: urate promoting heterogeneous nucleation of calcium oxalate in the crystalline phase, increasing calcium oxalate supersaturation and causing scaling in the ionic phase, or adsorbing onto urate crystal surfaces to drive crystal growth; a low urine pH environment can enhance this process. Furthermore, urate deposition in renal tubules under hyperuricemic conditions can cause oxidative stress and inflammatory injury, further promoting stone development (Moe and Xu, 2018; Miake et al., 2023). Although current understanding of the macroscopic processes of kidney stone formation, such as crystal nucleation, growth, aggregation, and adhesion to renal tubular epithelium, has progressed, the initiating molecular signaling events driving the excretion disorders of urinary lithogenic substances are not fully elucidated.

The calcium-sensing receptor (CaSR), a G protein-coupled receptor widely expressed in various segments of the renal tubules, is a core regulator of extracellular calcium ion homeostasis (Graca et al., 2016; Vezzoli et al., 2019). In the kidney, especially in the thick ascending limb (TAL), activation of CaSR has been confirmed to promote urinary calcium excretion through various mechanisms, such as upregulating the expression of the tight junction protein Claudin-14, thereby inhibiting reabsorption in renal tubular epithelial cells. This process is considered a “pro-lithogenic” pathway through which CaSR promotes calcium-containing stone formation (Lee et al., 2021; Luo et al., 2024). However, the function of CaSR is complex; in the collecting duct, it acidifies urine and inhibits water reabsorption to dilute lithogenic substances, exhibiting an “anti-lithogenic” aspect (Renkema et al., 2009; Procino et al., 2012). This functional duality suggests that the role of CaSR in the kidney stone formation network may be far more complex and refined than currently known.

On the other hand, the regulation of urinary oxalate concentration is crucial for calcium oxalate stone formation. Solute carrier family 26 member 6 (SLC26A6) is a key anion exchanger located on the apical membrane of renal proximal tubular epithelial cells, primarily responsible for mediating the secretion of oxalate from the cell into the lumen (Coe et al., 2005; Kere, 2006). Both clinical genetic studies and animal model experiments indicate that dysfunction of SLC26A6 leads to increased urinary oxalate excretion, significantly elevating the risk of calcium oxalate stones (Sancak et al., 2015; Singh et al., 2010). This establishes the central role of SLC26A6 in oxalate metabolic homeostasis.

A thought-provoking phenomenon is that clinical studies clearly confirm the frequent co-existence of hypercalciuria and hyperoxaluria (especially in COD/COM stone patients) (Gouru et al., 2018). This suggests a potential intrinsic crosstalk between the metabolic regulation of calcium and oxalate. Our preliminary experiments found that during stone formation, the expression of CaSR and SLC26A6 showed a synchronized upregulation trend. Therefore, we hypothesized that CaSR might act as an upstream regulatory molecule, influencing the expression and function of SLC26A6, thereby synergistically exacerbating the excretion disorders of urinary calcium and oxalate. However, the specific molecular bridge connecting these two key nodes, CaSR and SLC26A6, remains unknown.

As a G protein-coupled receptor, CaSR has diverse downstream signal transduction. Studies show that CaSR activation can increase intracellular cAMP levels, subsequently activating protein kinase A (PKA) (Macías-García et al., 2019). PKA, as an important serine/threonine kinase, widely participates in the regulation of cellular functions by phosphorylating downstream substrates. Notably, multiple studies confirm that the transport activity of SLC26A6 is positively regulated by the PKA signaling pathway (Tseng et al., 2020). Does PKA mediate the regulation of SLC26A6 by CaSR? Through bioinformatic analysis of the SLC26A6 promoter sequence, we predicted that Forkhead box protein O4 (FOXO4) is one of the transcription factors with the highest likelihood of binding to it. FOXO4 is a member of the FOXO transcription factor family, whose activity is regulated by phosphorylation by various kinases including PKA, and which translocates to the nucleus to perform transcriptional functions upon activation (Lu et al., 2021; Silveira et al., 2020).

Our group’s previous research confirmed that in a calcium oxalate kidney stone model, CaSR upregulates the expression of the tight junction protein Claudin-14 via the PKA-STAT3 pathway, thereby inhibiting the reabsorption of calcium ions in renal tubules and promoting hypercalciuria and stone formation (Luo et al., 2024). This discovery clarified the ‘pro-lithogenic’ role of CaSR in regulating calcium metabolism. However, given the frequent co-occurrence of hypercalciuria and hyperoxaluria in clinical practice, we speculated that the function of CaSR might be more complex. Therefore, this study aims to explore whether CaSR simultaneously regulates oxalate metabolism through another, yet unelucidated, signaling pathway. We propose the core hypothesis of this study: During experimental calcium oxalate kidney stone formation, calcium oxalate crystals (COM) functionally activate CaSR, initiating signal transduction that upregulates SLC26A6 expression via the CaSR-PKA-FOXO4 signaling cascade, ultimately leading to increased urinary oxalate excretion. This, synergizing with hypercalciuria, promotes the formation of calcium oxalate crystals.

This study comprehensively utilized *in vivo* animal models and *in vitro* cell models, combined with pharmacological interventions, molecular biology detection, and reporter gene analysis, aiming to systematically validate the existence of the CaSR-PKA-FOXO4-SLC26A6 signaling axis and its function in kidney stone formation. It is intended to provide experimental basis and theoretical support for a deeper understanding of the molecular pathogenesis of kidney stones and for finding new therapeutic targets.

## Materials and methods

### Materials

CaSR agonist R568 (GC17700), CaSR inhibitor NPS-2143 (GC16943), PKA agonist 8-Bromo-cAMP (GC16929), PKA inhibitor H-89 2HCl (GC10074), and FOXO4 inhibitor JY-2 were purchased from GlpBio Company. FOXO4 agonist TNF-α (GP20521) was purchased from GlpBio Company. Fetal bovine serum (FBS) and Dulbecco’s Modified Eagle Medium (DMEM) medium were purchased from Gibco Company. Rabbit polyclonal antibodies against CaSR (73303S) and Phospho-PKA Substrate (9624S) were purchased from Cell Signaling Technology Company. Rabbit polyclonal antibody against p-FOXO4 (Thr451) (AF39-1) was purchased from Affinity Company. Rabbit polyclonal antibody against SLC26A6 (sc-515930) was purchased from Santa Cruz Company. Mouse monoclonal antibody against β-actin (66009-1-Ig) and horseradish peroxidase (HRP)-labeled goat anti-rabbit/mouse IgG were purchased from Proteintech Company. BCA protein concentration assay kit and RIPA lysis buffer were purchased from Yeasen Biotechnology Co., Ltd. RNA extraction kit and reverse transcription kit were purchased from TransGen Biotech Co., Ltd. Real-time fluorescent quantitative PCR kit was purchased from Mei5 Biotechnology Co., Ltd. (Beijing). Dual-Luciferase® Reporter Assay System was purchased from Promega Company. Conventional chemical reagents such as ethylene glycol and ammonium chloride were purchased from Sigma-Aldrich or Sinopharm Chemical Reagent Co., Ltd.

### Cell culture and treatment

Rat renal tubular epithelial cells (NRK-52E) were purchased from Procell Life Science and Technology Co., Ltd. (Wuhan). NRK-52E cells were cultured in DMEM complete medium containing 10% FBS and 1% penicillin-streptomycin in a cell culture incubator at 37 °C with 5% CO_2_. Cells were passaged every 2–3 days, and cells in the logarithmic growth phase were used for experiments. Calcium oxalate monohydrate (COM) crystals were prepared according to classical literature methods (Chen et al., 2010). When cell confluence reached 70%–80%, the medium was replaced with serum-free medium for starvation treatment. Subsequently, according to experimental grouping, cells were co-cultured with COM crystals (134 μg/cm^2^) and the following drugs for 24 h: COM group (only COM crystals added); CaSR agonist (CaSR-a) group: COM + R568 (30 μM); CaSR inhibitor (CaSR-i) group: COM + NPS-2143 (10 μM); PKA agonist (PKA-a) group: COM + 8-Bromo-cAMP (30 μM); PKA inhibitor (PKA-i) group: COM + H-89 2HCl (30 μM); FOXO4 agonist (FOXO4-a) group: COM + TNF-α (20 ng/mL); FOXO4 inhibitor (FOXO4-i) group: COM + JY-2 (30 μM). Drug concentrations were determined through pre-experiments. After treatment, cells were collected for protein extraction.

### Experimental animals and protocol

This study protocol was approved by the Laboratory Animal Ethics Committee of Gannan Medical University. All animal procedures followed national regulations on laboratory animal welfare and ethics. Sixty healthy male Wistar rats were adaptively fed for 1 week under standard conditions and then randomly divided into six groups (n = 10) using a random number table: Blank Control (NC) group: Free access to distilled water, daily intramuscular injection of 0.1 mL saline (days 1–7), daily intragastric administration of 2 mL saline (days 24–30); Ethylene Glycol (E.G.,) group: Free access to 1% ethylene glycol (E.G.,) solution, daily intramuscular injection of 0.1 mL saline (days 1–7), daily intragastric administration of 1% ammonium chloride solution at 1 mL/kg (days 24–30); CaSR Agonist (CaSR-a) group: Free access to 1% E.G., solution, daily intramuscular injection of R568 (0.12 mg/kg) (days 1–7), daily intragastric administration of 1% ammonium chloride solution (same as, E.G., group); CaSR Inhibitor (CaSR-i) group: Free access to 1% E.G., solution, daily intramuscular injection of NPS-2143 (0.15 mg/kg) (days 1–7), daily intragastric administration of 1% ammonium chloride solution; PKA Inhibitor (PKA-i) group: Free access to 1% E.G., solution, daily intramuscular injection of H-89 2HCl (0.5 mg/kg) (days 1–7), daily intragastric administration of 1% ammonium chloride solution; FOXO4 Inhibitor (FOXO4-i) group: Free access to 1% E.G., solution, daily intramuscular injection of JY-2 (5 mg/kg) (days 1–7), daily intragastric administration of 1% ammonium chloride solution. All drug injection concentrations were determined based on instructions and pre-experimental results. On day 29 of the experiment, 24-h urine was collected. At the end of the experiment, all rats were anesthetized by intraperitoneal injection of sodium pentobarbital (50 mg/kg) and sacrificed by cervical dislocation. Both kidneys were rapidly dissected, weighed to calculate the kidney-to-body weight ratio. Part of the tissue from one kidney was flash-frozen in liquid nitrogen and transferred to a −80 °C freezer for protein and RNA extraction, while another part was fixed in 10% formalin solution for paraffin section preparation.

### Pathological examination

Formalin-fixed kidney tissues were dehydrated, cleared, embedded in paraffin, and sectioned into 4 μm thick slices. After deparaffinization and hydration, sections were stained with Hematoxylin and Eosin (HE) and Pizzolato’s staining for calcium. Stained sections were observed under light microscopy and polarized light microscopy for renal histomorphological changes and crystal deposition.

### Western blotting analysis

Total protein was extracted from kidney tissues or cells using RIPA lysis buffer containing protease and phosphatase inhibitors. Protein concentration was determined by the BCA method and adjusted to a uniform concentration. Protein samples were separated by SDS-PAGE gel electrophoresis and transferred to PVDF membranes. After blocking with 5% skim milk at room temperature for 2 h, the membranes were incubated with corresponding primary antibodies: CaSR (1:2500), p-PKA Substrate (1:1000), p-FOXO4 (Thr451) (1:1000), SLC26A6 (1:1000), β-actin (1:5000) at 4 °C overnight. After TBST washing, membranes were incubated with corresponding HRP-labeled secondary antibodies (1:2500) at room temperature for 2 h. Finally, signals were developed using an ECL chemiluminescence kit on an imaging system. Band grayscale values were analyzed using ImageJ software, and the relative expression was represented by the ratio of the target protein grayscale value to that of β-actin.

### Real-time fluorescence quantitative PCR (RT-qPCR)

Total RNA was extracted from kidney tissues using an RNA extraction kit and reverse-transcribed into cDNA. Using cDNA as a template, amplification was performed on a real-time fluorescent quantitative PCR instrument using Top Green qPCR SuperMix. The reaction program was: 95 °C pre-denaturation for 3 min; 40 cycles of 95 °C for 5 s, 60 °C for 30 s, 72 °C for 10 s. Primer sequences were as follows: β-actin: Forward 5′-ACC​ACA​GTC​CAT​GCC​ATC​AC-3′, Reverse 5′-TCC​ACC​ACC​CTG​TTG​CTG​TA-3'; CaSR: Forward 5′-ACC​TTT​ACC​TGT​CCC​CTG​AA-3′, Reverse 5′-GGG​CAA​CAA​AAC​TCA​AGG​TG-3'; SLC26A6: Forward 5′-ATG​TAC​TTC​GCC​AAC​GCT​GA-3′, Reverse 5′-TTT​TCT​GGG​TGA​TGA​GGC​GG-3'. The relative expression level of gene mRNA was calculated using the 2^–ΔΔCt method.

### Immunohistochemistry

Paraffin sections were deparaffinized, hydrated, and subjected to antigen retrieval using citrate sodium buffer (pH 6.0). Subsequently, endogenous peroxidase activity was blocked with 3% H_2_O_2_ solution. Then, non-specific binding sites were blocked with 5% BSA. Sections were incubated with primary antibodies CaSR (1:500), p-PKA Substrate (1:600), p-FOXO4 (1:300), SLC26A6 (1:300) in a humidified chamber at 4 °C overnight. After PBS washing, corresponding HRP-labeled polymer secondary antibodies were added and incubated at 37 °C for 30 min. Color development was performed using DAB substrate, nuclei were counterstained with hematoxylin, and finally, sections were dehydrated, cleared, and mounted with neutral balsam. Images were observed and captured under a light microscope.

### Dual-luciferase reporter gene assay

The rat SLC26A6 gene promoter sequence (approximately 2000 bp) was cloned into the pGL4.1 luciferase reporter vector using molecular cloning technology. The recombinant plasmid and the internal control plasmid pRL-TK were co-transfected into well-growing NRK-52E cells using TransIntro EL transfection reagent. After 6 h of transfection, the medium was replaced with complete medium. Twenty-four hours after transfection, cells were divided into three groups: negative control group, FOXO4 agonist group (TNF-α, 20 ng/mL), and FOXO4 inhibitor group (JY-2, 30 μM), and cultured for another 24 h. Cells were collected, and according to the instructions of the dual-luciferase reporter gene detection kit, firefly and Renilla luciferase activities were measured using a multifunctional microplate reader. The relative activity of the reporter gene was expressed as the ratio of firefly luciferase activity to Renilla luciferase activity.

### Statistical analysis

All data are expressed as mean ± standard deviation. Statistical analysis was performed using SPSS 22.0 software. Data were first tested for normality. Comparisons between two groups were performed using independent samples t-test. Comparisons among multiple groups were performed using one-way analysis of variance (ANOVA), followed by post-hoc tests (such as LSD test). P < 0.05 was considered statistically significant.

## Results

### Calcium oxalate crystals upregulate CaSR and SLC26A6 expression *in vivo* and *in vitro*

From a physicochemical perspective, the precipitation of calcium oxalate crystals in urine depends on whether its ion activity product reaches supersaturation. For calcium oxalate monohydrate (COM), this critical value is approximately 4 × 10^–9 (Daudon et al., 2016). Normal human urine under physiological conditions is usually below this threshold. However, when metabolic disorders such as hyperoxaluria occur, the saturation of calcium oxalate in urine significantly increases and surpasses this critical point, thereby driving crystal nucleation and precipitation. This forms the theoretical basis for successfully establishing a rat kidney stone model using ethylene glycol to induce hyperoxaluria. Studies further indicate that the oxidative stress triggered by COM crystals can damage renal tubular epithelial cells, thereby promoting crystal aggregation, retention, and eventual stone formation (Thamilselvan et al., 2014; Khan, 2006).

**FIGURE 1:**
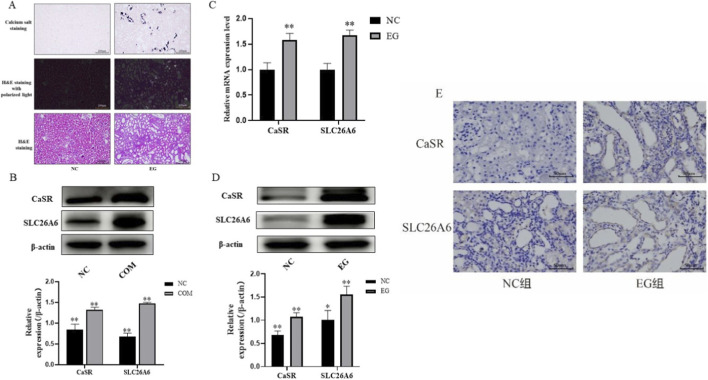
Calcium oxalate crystals upregulate CaSR and SLC26A6 expression *in vivo* and *in vitro*. **(A)** Representative images of renal tissue pathological sections from each group of rats (HE staining, Pizzolato’s staining, and polarized light microscopy). **(B)** Western blotting detection of CaSR and SLC26A6 protein expression levels in NRK-52E cells after COM crystal intervention. **(C,D)** RT-qPCR **(C)** and Western blotting **(D)** detection of CaSR and SLC26A6 mRNA and protein expression levels in renal tissues of each group of rats. **(E)** Immunohistochemical analysis revealed the tubular localization patterns of CaSR and SLC26A6 in rat kidney tissue. Low-power field images demonstrated their widespread distribution within both the renal cortex and medulla. Precise cellular colocalization could only be assessed through high-power confocal analysis combined with segment-specific markers, representing a limitation of this study. Data are presented as mean ± standard deviation, *p < 0.05, **p < 0.01 vs. NC group or Control group.

Based on the above principles, we successfully established an ethylene glycol-induced rat kidney stone model. As shown in Figure 1A, compared to the structurally normal NC group, the kidneys of the, E.G., group rats showed significant damage, including renal tubular epithelial cell edema, disordered arrangement, and inflammatory cell infiltration; both polarized light microscopy and specific calcium salt staining confirmed the presence of numerous calcium oxalate crystal deposits in the renal tubules, thus validating the successful model establishment.

On this model basis, we detected changes in CaSR and SLC26A6 expression. *In vitro*, compared to the control group, COM crystal treatment significantly upregulated the protein expression levels of CaSR and SLC26A6 in rat renal tubular epithelial cells (NRK-52E) (Figure 1B). *In vivo*, we found that the mRNA (Figure 1C) and protein (Figure 1D) expression levels of CaSR and SLC26A6 in the kidney tissues of the model group (E.G.,) were significantly higher than those in the blank control group (NC). These quantitative detection results based on total tissue homogenates consistently indicate that after calcium oxalate crystal treatment, the mRNA and protein expression levels of both CaSR and SLC26A6 are significantly increased.

Furthermore, we observed the spatial distribution of these proteins in renal tissues through immunohistochemical staining. As shown in Figure 1E, CaSR and SLC26A6 were mainly localized in renal tubular epithelial cells. Although we observed a trend of darker staining in the, E.G., group, due to potential differences in the basal expression levels of proteins in the cortical and medullary regions, this result serves only as a qualitative reference for protein localization. Our core conclusion regarding the upregulation of CaSR and SLC26A6 expression is primarily based on the aforementioned unbiased quantitative detection methods (Western blotting and qPCR) of tissue regions.

### CaSR regulates SLC26A6 expression via the transcription factor FOXO4

**FIGURE 2:**
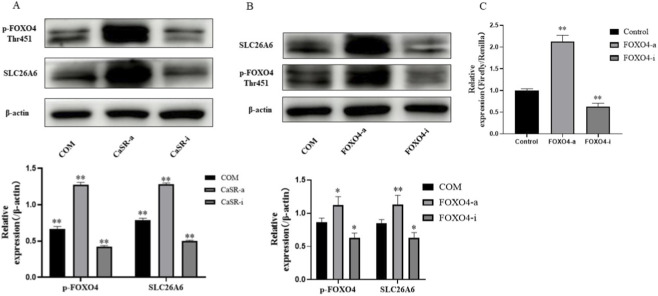
CaSR regulates SLC26A6 expression via the transcription factor FOXO4. **(A)** Western blotting detection of p-FOXO4 (Thr451) and SLC26A6 protein expression levels in NRK-52E cells treated with CaSR agonist (R568, 30 μM) or inhibitor (NPS-2143, 10 μM) in the presence of COM crystals. **(B)** Western blotting detection of SLC26A6 protein expression levels in NRK-52E cells treated with FOXO4 agonist (TNF-α, 20 ng/mL) or inhibitor (JY-2, 30 μM) in the presence of COM crystals. **(C)** Dual-luciferase reporter gene assay detecting the effect of FOXO4 on SLC26A6 promoter activity. Data are presented as mean ± standard deviation, *p < 0.05, **p < 0.01 vs. COM group; #p < 0.05, ##p < 0.01 vs. specified group.

The above results indicated a positive correlation between CaSR and SLC26A6 expression, leading us to hypothesize that CaSR might be an upstream regulator of SLC26A6. To test this hypothesis, we treated NRK-52E cells with CaSR agonist (R568) and inhibitor (NPS-2143) in the presence of COM crystals. The results showed that activating CaSR further significantly increased SLC26A6 protein expression levels, whereas inhibiting CaSR effectively blocked the COM-induced upregulation of SLC26A6 expression (Figure 2A). Concurrently, the phosphorylation level of FOXO4 showed the same trend of change (Figure 2A), suggesting that FOXO4 might mediate CaSR signal transmission.

To confirm FOXO4 function, we directly modulated its activity. Treatment with a FOXO4-specific agonist (TNF-α) significantly upregulated SLC26A6 protein expression, whereas treatment with a FOXO4 inhibitor (JY-2) significantly suppressed its expression (Figure 2B). To further establish FOXO4 as a transcriptional regulator of SLC26A6, we performed a dual-luciferase reporter assay. Following transfection with the reporter vector containing the SLC26A6 promoter, we found that FOXO4 activation significantly increased promoter activity, while inhibition substantially decreased it (Figure 2C). These results demonstrate that FOXO4 functionally regulates SLC26A6 promoter activity.

### CaSR promotes FOXO4 phosphorylation via the PKA signaling pathway

**FIGURE 3:**
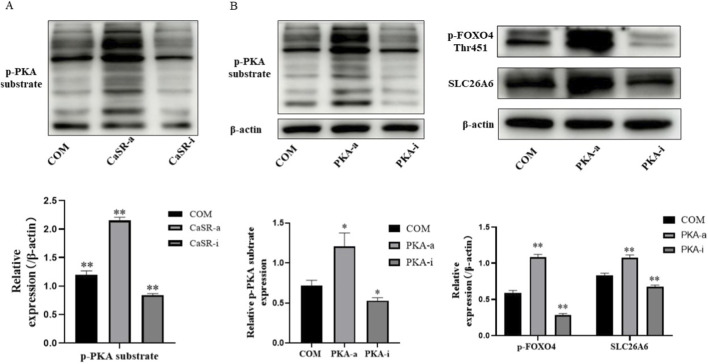
CaSR promotes FOXO4 phosphorylation via the PKA signaling pathway. **(A)** Western blotting detection of p-PKA substrate and p-FOXO4 (Thr451) protein expression levels in NRK-52E cells treated with CaSR agonist (R568, 30 μM) or inhibitor (NPS-2143, 10 μM) in the presence of COM crystals. **(B)** Western blotting detection of p-FOXO4 (Thr451) and SLC26A6 protein expression levels in NRK-52E cells treated with PKA agonist (8-Bromo-cAMP, 30 μM) or inhibitor (H-89 2HCl, 30 μM) in the presence of COM crystals. Data are presented as mean ± standard deviation, *p < 0.05, **p < 0.01 vs. COM group; #p < 0.05, ##p < 0.01 vs. specified group.

After clarifying the CaSR-FOXO4-SLC26A6 regulatory axis, we further investigated the intracellular signal connecting CaSR and FOXO4. By detecting PKA substrate phosphorylation levels via Western blotting, we confirmed in NRK-52E cells that activating CaSR significantly enhanced PKA activity. Inhibiting CaSR produced the opposite effect (Figure 3A). This indicates that in the stone microenvironment, CaSR activates the cAMP/PKA signaling pathway.

Does PKA mediate the phosphorylation of FOXO4 and the subsequent expression of SLC26A6? We validated this using a PKA-specific agonist (8-Bromo-cAMP) and inhibitor (H-89 2HCl). The results showed that directly activating PKA significantly enhanced FOXO4 phosphorylation and upregulated SLC26A6 expression; whereas inhibiting PKA attenuated FOXO4 phosphorylation and downregulated SLC26A6 expression (Figure 3B). These data indicate that PKA is the key signaling molecule downstream of CaSR regulating FOXO4 and SLC26A6.

### Intervention on CaSR, PKA, or FOXO4 inhibits experimental kidney stone formation

**FIGURE 4:**
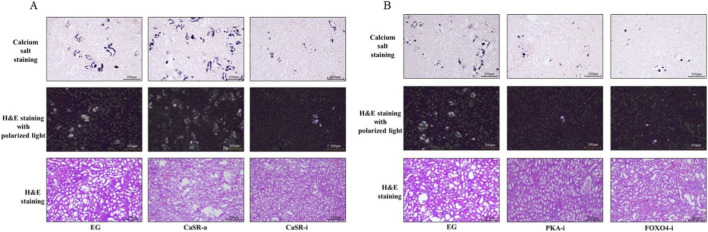
Crystal deposition and tissue damage in the kidneys of rats from each group **(A)** Hematoxylin and eosin (HE) staining, polarized light microscopy, and Pizzolato’s calcium salt staining of kidney tissue from rats in the EG, CaSR-a, and CaSR-i groups. HE staining reveals tubular architecture and the extent of damage; polarized light microscopy reveals the birefringence of calcium oxalate crystals; Pizzolato’s staining stains calcium oxalate crystals black. **(B)** Hematoxylin and eosin (HE) staining, polarized light microscopy, and Pizzolato’s calcium salt staining of renal tissues from rats in the EG, PKA-i, and FOXO4-i groups. HE staining reveals the extent of tubular damage in each group; polarized light microscopy and calcium salt staining jointly confirm differences in crystal deposition among the groups.

To verify the physiological significance of the above molecular pathway at the whole animal level, we conducted intervention studies in the ethylene glycol-induced rat kidney stone model. Renal histopathological examination showed that compared to the, E.G., alone group, activating CaSR (CaSR-a group) significantly exacerbated crystal deposition and tissue damage within the renal tubules; whereas inhibiting CaSR (CaSR-i group), PKA (PKA-i group), or FOXO4 (FOXO4-i group) all effectively alleviated stone formation (Figures 4A,B).

**FIGURE 5:**
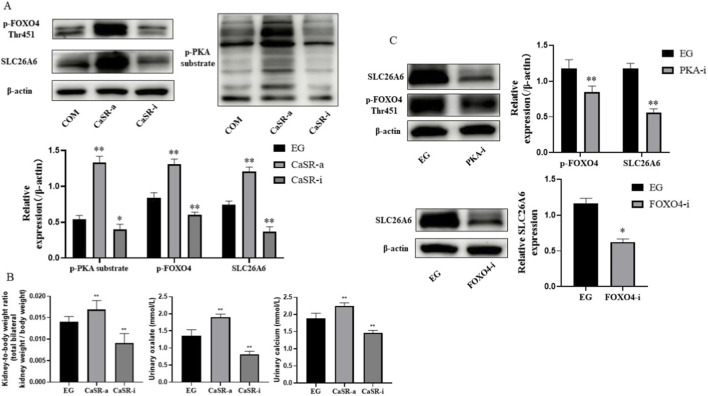
Effects of intervening in the CaSR-PKA-FOXO4 signaling axis on urinary biochemical indicators and renal protein expression in rats. **(A)** Western blotting detection of p-PKA substrate, p-FOXO4 (Thr451), and SLC26A6 protein expression levels in renal tissues of each group of rats. **(B)** Concentrations of calcium ions and oxalate in 24-h urine of each group of rats. **(C)** Quantitative analysis of SLC26A6 protein expression from **(A)**. Data are presented as mean ± standard deviation, *p < 0.05, **p < 0.01 vs. E.G., group.

Molecular and biochemical analyses showed that the CaSR-a group had the highest protein expression levels of p-PKA substrate, p-FOXO4, and SLC26A6 in the kidneys (Figures 5A,C), as well as the highest urinary calcium and oxalate concentrations (Figure 5B). In contrast, the CaSR-i, PKA-i, and FOXO4-i groups all reduced SLC26A6 protein expression (Figures 5A,C) and urinary oxalate excretion (Figure 5B) to varying degrees. Our in-depth analysis of urinary biochemical indicators found that in the CaSR-a group, the increase in urinary oxalate (approximately 40.1%) was greater than the increase in urinary calcium (approximately 19.8%); correspondingly, in the CaSR-i group, the decrease in urinary oxalate (approximately 40.3%) was also much greater than the decrease in urinary calcium (approximately 21.9%). These *in vivo* experimental data indicate that intervening on CaSR, PKA, or FOXO4 *in vivo* can inhibit SLC26A6 expression and reduce stone formation, and that regulating CaSR has a particularly significant impact on urinary oxalate excretion, further supporting the important role of the CaSR-PKA-FOXO4-SLC26A6 signaling axis in calcium oxalate kidney stone formation.

## Discussion

The mechanism of kidney stone formation is complex, with urinary calcium oxalate supersaturation being a key initiating factor. This study is the first to systematically reveal the core role of a signaling pathway composed of CaSR-PKA-FOXO4-SLC26A6 in experimental calcium oxalate kidney stone formation.

An important consideration emerging from our study concerns the anatomical relationship between CaSR and SLC26A6 and the validation strategy of our findings.While CaSR is predominantly expressed in the thick ascending limb and collecting duct, and SLC26A6 is localized to the proximal tubule under physiological conditions, our study provides evidence for functional regulation at two distinct levels, employing a hierarchical validation strategy that distinguishes between molecular mechanism and anatomical localization: At the cellular level, using NRK-52E cells, we establish the molecular feasibility of the CaSR-PKA-FOXO4-SLC26A6 signaling cascade, demonstrating that these components can form a functional signaling axis within renal tubular epithelial cells. At the organismal level, our *in vivo* experiments demonstrate that pharmacological modulation of CaSR consistently regulates SLC26A6 expression and urinary oxalate excretion during stone formation. This tiered approach is standard in molecular pharmacology: first establishing that a pathway exists and operates (cellular level), then confirming its functional significance under pathological conditions (organismal level), before finally dissecting anatomical specificity.

It is crucial to note that our data establish functional connectivity between CaSR activation and SLC26A6 upregulation, but do not definitively distinguish between potential mechanisms of signal transmission: direct cell-autonomous signaling, which would require co-expression of CaSR and SLC26A6 in the same cells (potentially occurring in specific pathological contexts or cellular subpopulations); or indirect paracrine/endocrine mechanisms, whereby CaSR activation in distal nephron segments influences proximal tubular SLC26A6 through secreted mediators or systemic hormonal changes. The convergence of pharmacological effects at multiple pathway nodes (CaSR, PKA, FOXO4) supports the existence of this regulatory axis, regardless of the precise anatomical route.

At the mechanistic level, one of the most critical findings of this study is identifying the pivotal role of the transcription factor FOXO4 in this pathway and revealing the intermediary role of PKA.We confirmed through multiple sets of experiments that FOXO4 is the bridge connecting CaSR and SLC26A6.On one hand, CaSR activity is an upstream event regulating FOXO4 phosphorylation (Thr451) and its transcriptional function (Figure 2A). On the other hand, directly intervening in FOXO4 activity is sufficient to bidirectionally regulate SLC26A6 protein expression (Figure 2B). Particularly important, the dual-luciferase reporter gene experiment demonstrated that FOXO4 functionally regulates SLC26A6 promoter activity, consistent with its role as a transcriptional regulator of SLC26A6 expression. While these findings strongly support FOXO4-mediated transcriptional control, definitive proof of direct physical binding at the endogenous chromatin level requires chromatin immunoprecipitation (ChIP) assays, which represent an important direction for future mechanistic studies.As a G protein-coupled receptor, the diversity of CaSR’s downstream signals is the basis for its multiple functions. This study focused on the cAMP/PKA pathway. We confirmed that CaSR activation significantly increased the phosphorylation level of PKA substrates (Figure 3A). More importantly, we found that PKA is the key upstream kinase for FOXO4 phosphorylation: activating PKA successfully mimicked the effect of CaSR activation, while inhibiting PKA effectively blocked the regulation of FOXO4 and SLC26A6 by CaSR (Figure 3B). These results clearly outline the signaling axis of CaSR -cAMP/PKA -FOXO4.

**FIGURE 6:**
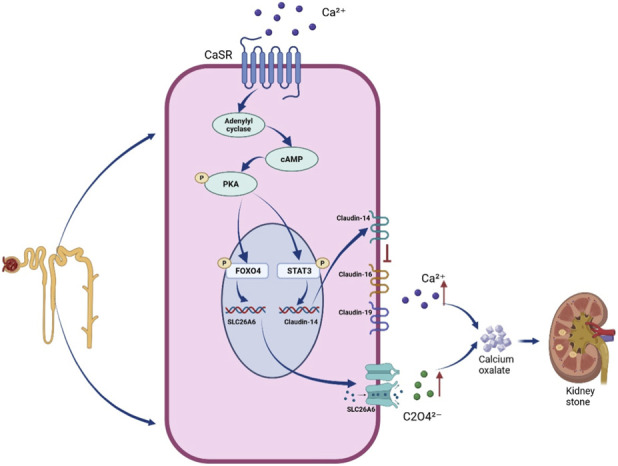
Schematic diagram of the mechanism by which CaSR synergistically promotes calcium oxalate stone formation through dual signaling pathways. Under stimulation by calcium oxalate crystals (COM), the calcium-sensing receptor (CaSR) on the membrane of renal tubular epithelial cells is activated. The activated CaSR activates protein kinase A (PKA) via G proteins. PKA then functions through two parallel pathways: Phosphorylates Signal Transducer and Activator of Transcription 3 (STAT3), causing its nuclear translocation and upregulation of the tight junction protein Claudin-14 expression. Claudin-14 inhibits paracellular calcium reabsorption, leading to hypercalciuria; Phosphorylates Forkhead box protein O4 (FOXO4), causing its nuclear translocation and binding to the SLC26A6 gene promoter, upregulating its expression and promoting oxalate secretion into the lumen, leading to hyperoxaluria. The increased excretion of urinary calcium and oxalate collectively exacerbates the supersaturation of calcium oxalate in urine, ultimately promoting kidney stone formation.

This study elucidates a novel signaling pathway regulating oxalate metabolism. Combined with our group’s previous finding--that CaSR regulates Claudin-14 expression via the PKA-STAT3 pathway, thereby affecting urinary calcium excretion (Luo et al., 2024) -- it is now clear that CaSR, as a key upstream sensor, activates two parallel downstream pathways during kidney stone formation, coordinately regulating the excretion of these two key lithogenic substances, urinary calcium and urinary oxalate.We draw this conclusion based on the following two key pieces of evidence: First, in this study, activating CaSR (CaSR-a group) simultaneously increased urinary calcium and oxalate excretion, and the increase in urinary oxalate (40.1%) was greater than that in urinary calcium (19.8%); conversely, inhibiting this pathway (CaSR-i group) showed the opposite trend, and the decrease in urinary oxalate (40.3%) was also much greater than that in urinary calcium (21.9%) (Figure 5B). This suggests that this pathway plays a dominant role in regulating oxalate metabolism, and its contribution to the stone phenotype may be particularly prominent; Second, indirect evidence from previous studies is highly enlightening. In Cldn14 gene knockout rats, although specific regulation of CaSR could no longer affect stone formation, ethylene glycol could still successfully induce stones. The traditional explanation for this phenomenon is the compensatory effect of other Claudin family members. However, from the new perspective revealed by this study, a reasonable supplementary explanation is that after the calcium metabolism pathway was blocked by ‘gene knockout’, ethylene glycol might have activated the newly discovered CaSR-PKA-FOXO4-SLC26A6 ‘oxalate pathway’ we identified, driving hyperoxaluria and thereby initiating stone formation. These two studies corroborate each other, jointly outlining a more comprehensive regulatory network of CaSR in stone formation (Figure 6).

The functional significance of CaSR-SLC26A6 co-expression in proximal tubular epithelial cells extends beyond simple transcriptional regulation.We propose that this co-expression establishes an autocrine signaling mechanism with important pathophysiological implications for stone formation.Under hyperoxaluric conditions, COM crystals forming within the proximal tubular lumen can activate apically-localized CaSR, triggering the PKA-FOXO4 cascade and upregulating SLC26A6. This leads to enhanced oxalate secretion into the tubular fluid, creating a feed-forward mechanism that exacerbates calcium oxalate supersaturation and promotes further crystal nucleation and growth.This mechanism may be particularly relevant during episodes of acute hyperoxaluria. From a therapeutic perspective, local inhibition of proximal tubular CaSR or downstream PKA/FOXO4 signaling could potentially interrupt this feed-forward loop, representing a novel strategy for reducing urinary oxalate excretion and stone formation risk.

While our data support an intracellular direct signaling cascade linking CaSR to SLC26A6 *in vitro*, we recognize that inter-segmental paracrine or systemic endocrine mechanisms may also contribute to coordinated regulation *in vivo*. CaSR activation in the thick ascending limb or collecting duct could modulate tubular fluid composition (calcium concentration, pH, osmolarity) that indirectly affects SLC26A6 activity in downstream proximal tubular segments. Additionally, systemic hormones regulated by CaSR signaling, such as parathyroid hormone and active vitamin D, may indirectly influence oxalate metabolism through proximal tubular mechanisms that intersect with our identified PKA-FOXO4 pathway. Future studies employing nephron segment-specific gene knockout models or kidney microperfusion techniques will be essential to dissect these complex inter-segmental interactions.

This study has several limitations that warrant clarification. First, the results were obtained exclusively in male Wistar rats and require validation in human kidney tissue and clinical populations, with consideration of sex differences in future research. Second, the use of whole kidney homogenate samples precludes the precise identification of the specific nephron segments where CaSR-mediated regulation of SLC26A6 occurs. Although our immunohistochemical data support its primary localization in renal tubular epithelial cells, current methods cannot determine pathway activation in specific segments. Future studies employing laser capture microdissection, single-cell RNA sequencing, or immunomagnetic isolation of specific tubular segments are required to precisely define the nephron segment(s) involved in this pathway. Third, while pharmacological interventions offer valuable functional insights, genetic interventions-such as siRNA-mediated knockdown or CRISPR/Cas9-mediated gene editing-can provide more definitive causal evidence by eliminating potential off-target effects. Fourth, while our reporter gene assay indicates FOXO4 functionally regulates SLC26A6 promoter activity, confirming direct physical binding and identifying specific FOXO4 binding sites within the SLC26A6 promoter requires chromatin immunoprecipitation (ChIP) and potentially ChIP-seq analysis. These represent key directions for future mechanistic studies. Fifth, although our data indicate that COM crystals can activate the CaSR signaling pathway, the precise molecular mechanism of this activation-whether through direct ligand-receptor interaction, membrane-mediated mechanical transduction, or indirect secondary signaling events-remains to be definitively established.

In summary, this study systematically elucidates a novel signaling pathway activated during calcium oxalate kidney stone formation: Renal tubular epithelial cells perceive calcium oxalate crystal stimulation via CaSR, activate the transcription factor FOXO4 through PKA signaling, which then upregulates SLC26A6 expression, ultimately promoting urinary oxalate excretion and accelerating stone formation. This discovery, parallel to the known CaSR-Claudin-14 calcium metabolism pathway, greatly enriches the understanding of the regulatory network of renal ion metabolic homeostasis and provides a new theoretical basis and experimental foundation for the etiological prevention of kidney stones and the development of targeted therapeutic strategies.

## Data Availability

The original contributions presented in the study are included in the article/supplementary material, further inquiries can be directed to the corresponding authors.
